# Supplementary material for: N-Glycosylation of TREK-1/hK2P2.1 Two-Pore-Domain Potassium (K2P) Channels
Source: Int J Mol Sci. 2019 Oct 20;20(20):5193. doi: 10.3390/ijms20205193 (PMC6829520; doi:10.3390/ijms20205193)

Supplementary figure 1

Western blot: Tunicamycin treatment of TREK-1, expressed in *Xenopus* oocytes: (from figure 2a)

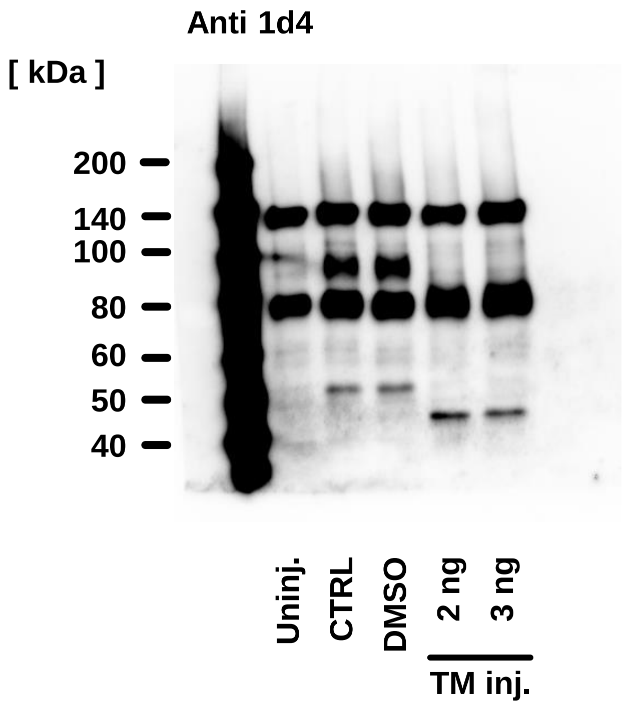

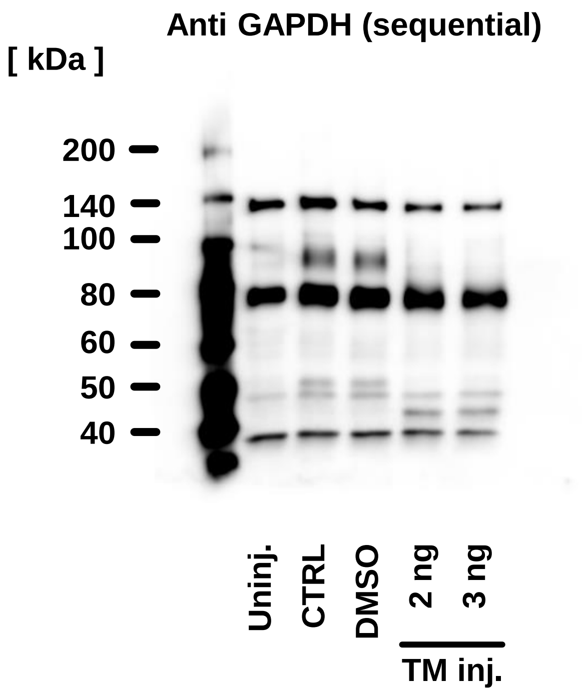

Supplementary figure 2

Western blot: Glycosylation deficient TREK-1 mutants, expressed in *Xenopus* oocytes: (from figure 3a)

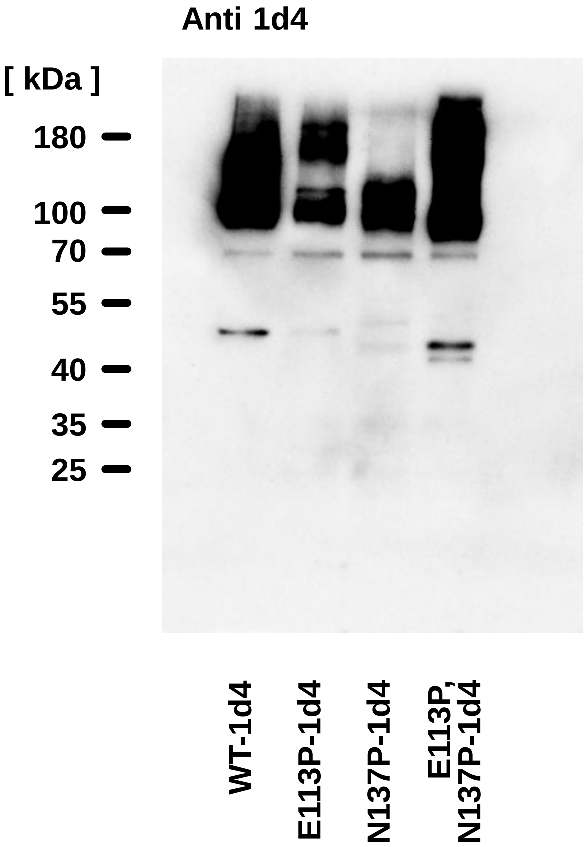

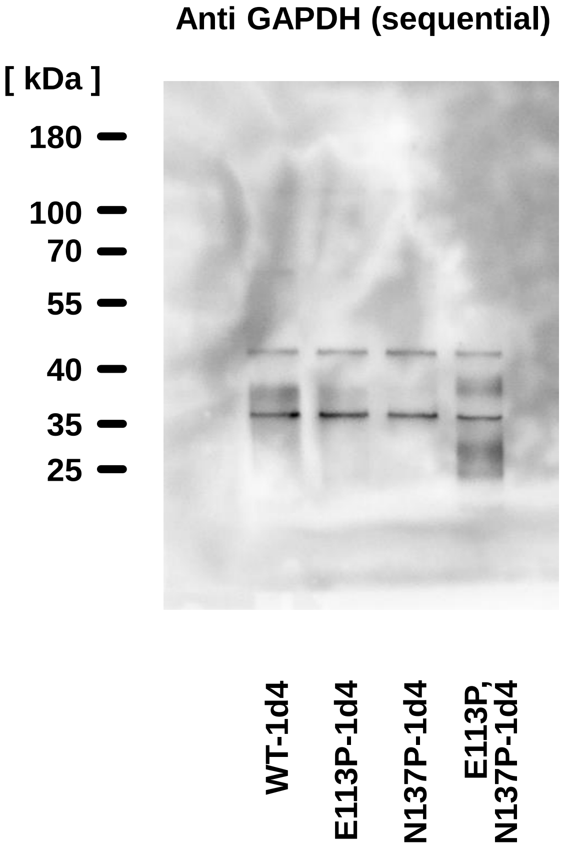

Supplementary figure 3

Western blot: Glycosylation deficient TREK-1 mutants, expressed in *Xenopus* oocytes: (from figure 3b)

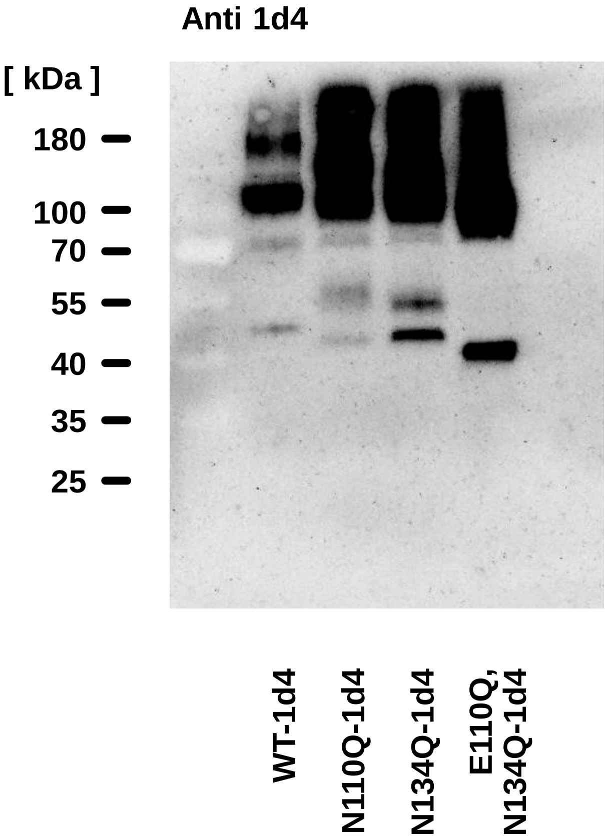

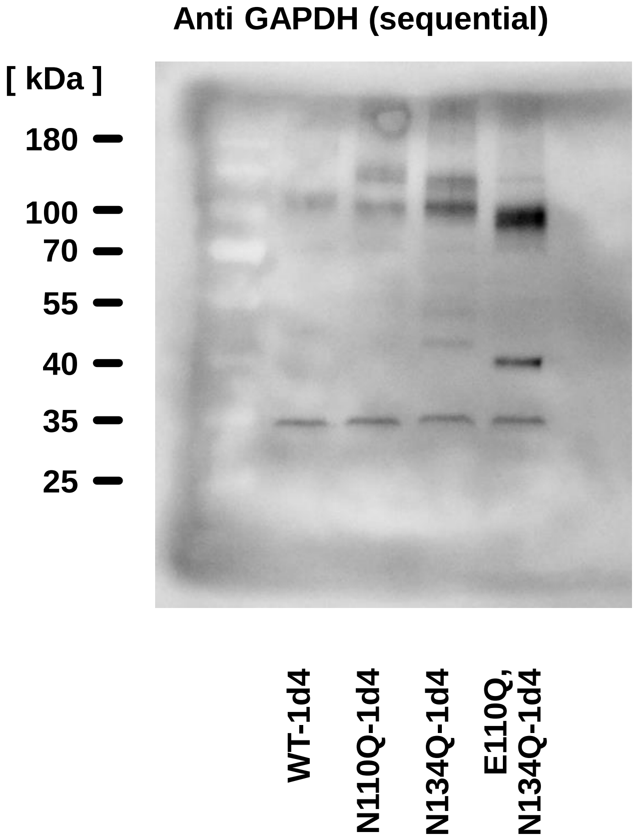

Supplementary figure 4

Western blot: PNGase F and tunicamycin treatment of TREK-1, expressed in HEK 293T cells: (from figure 4a)

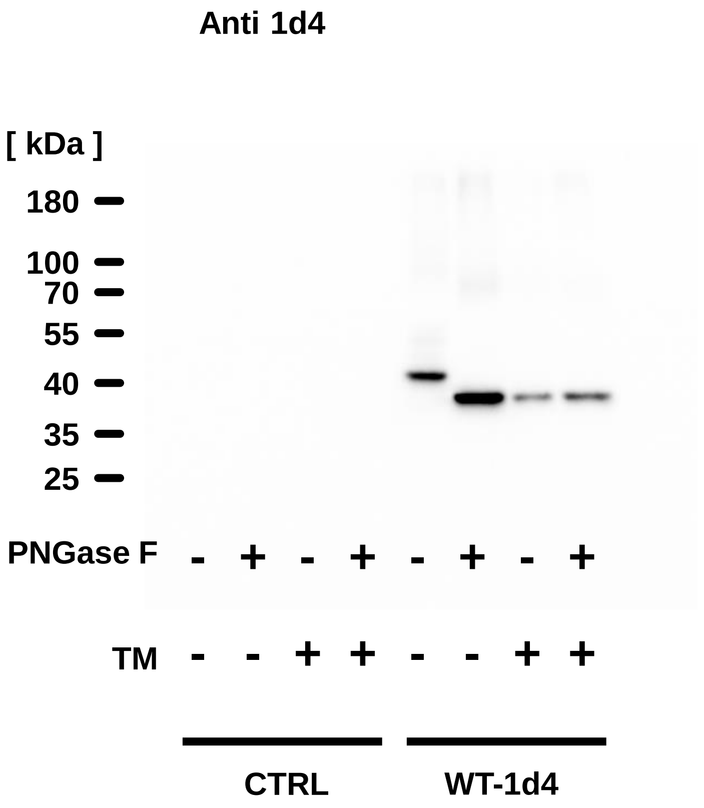

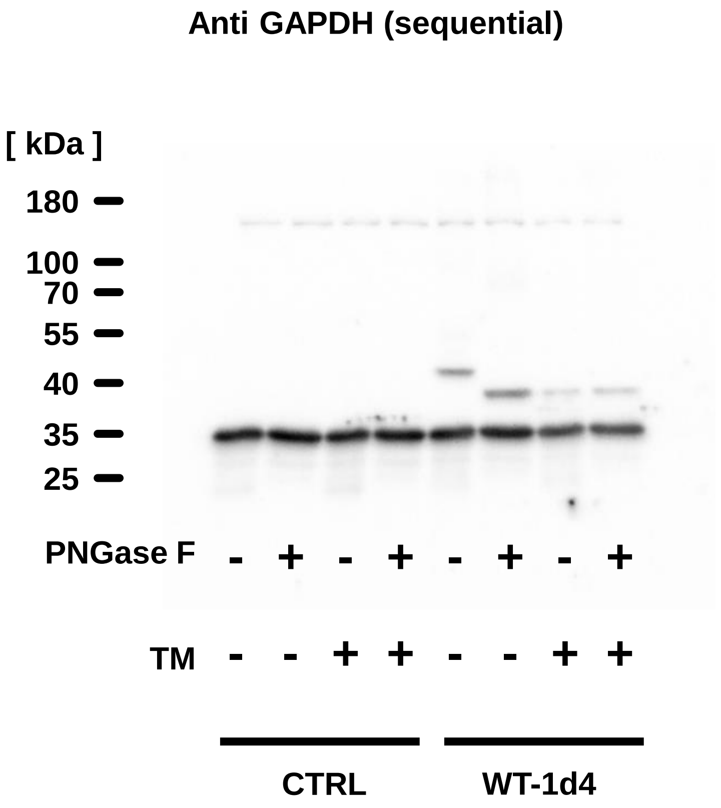

Supplementary figure 5

Western blot: Glycosylation deficient TREK-1 mutants, expressed in HEK 293T cells: (from figure 4b)

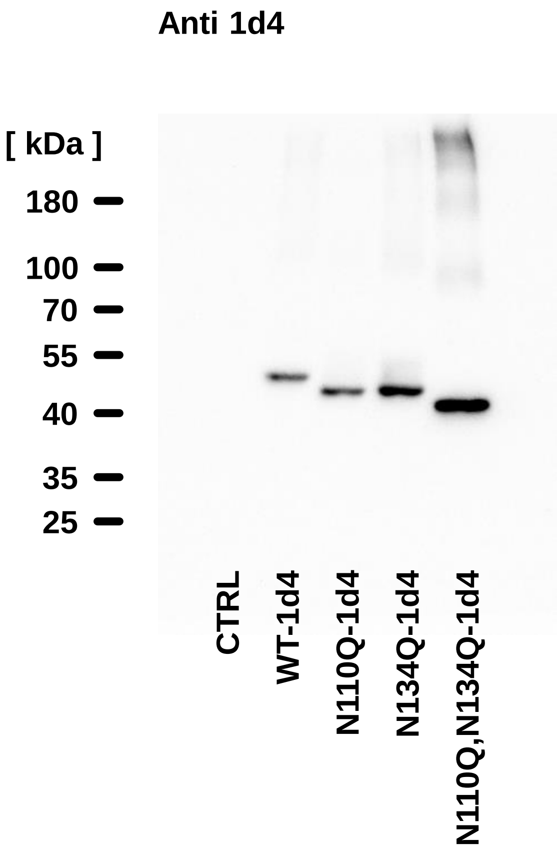

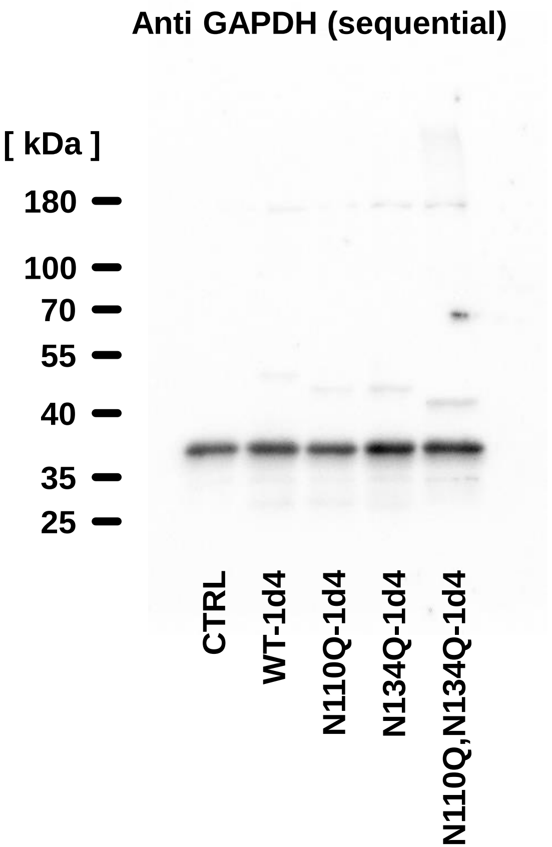

Supplementary figure 6

Western blot: PNGase F and tunicamycin treatment of glycosylation deficient TREK-1 mutants: (from figure 4c)

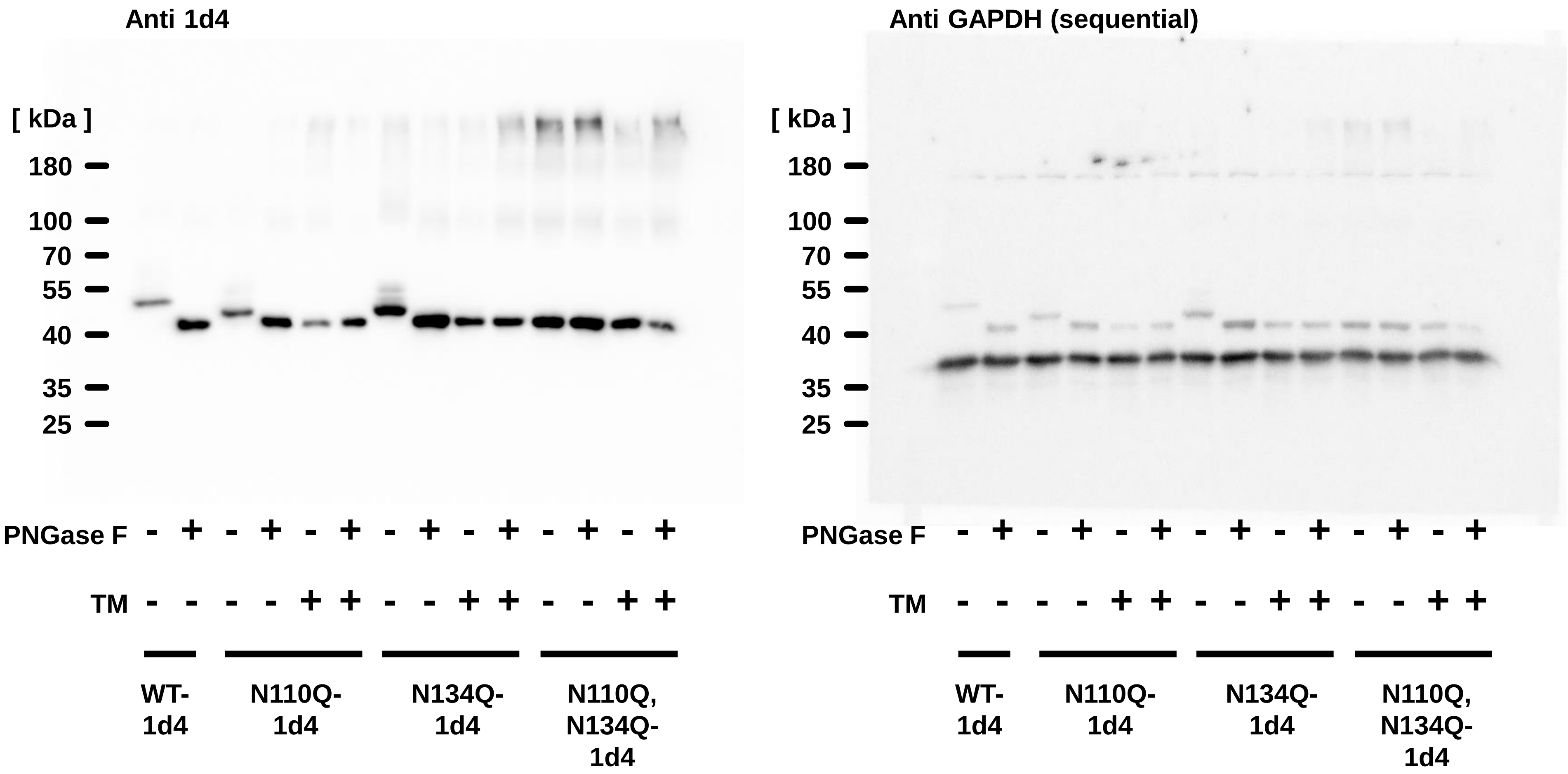

Supplementary figure 7

Western blot membrane 1: Surface expression (from figure 5b):

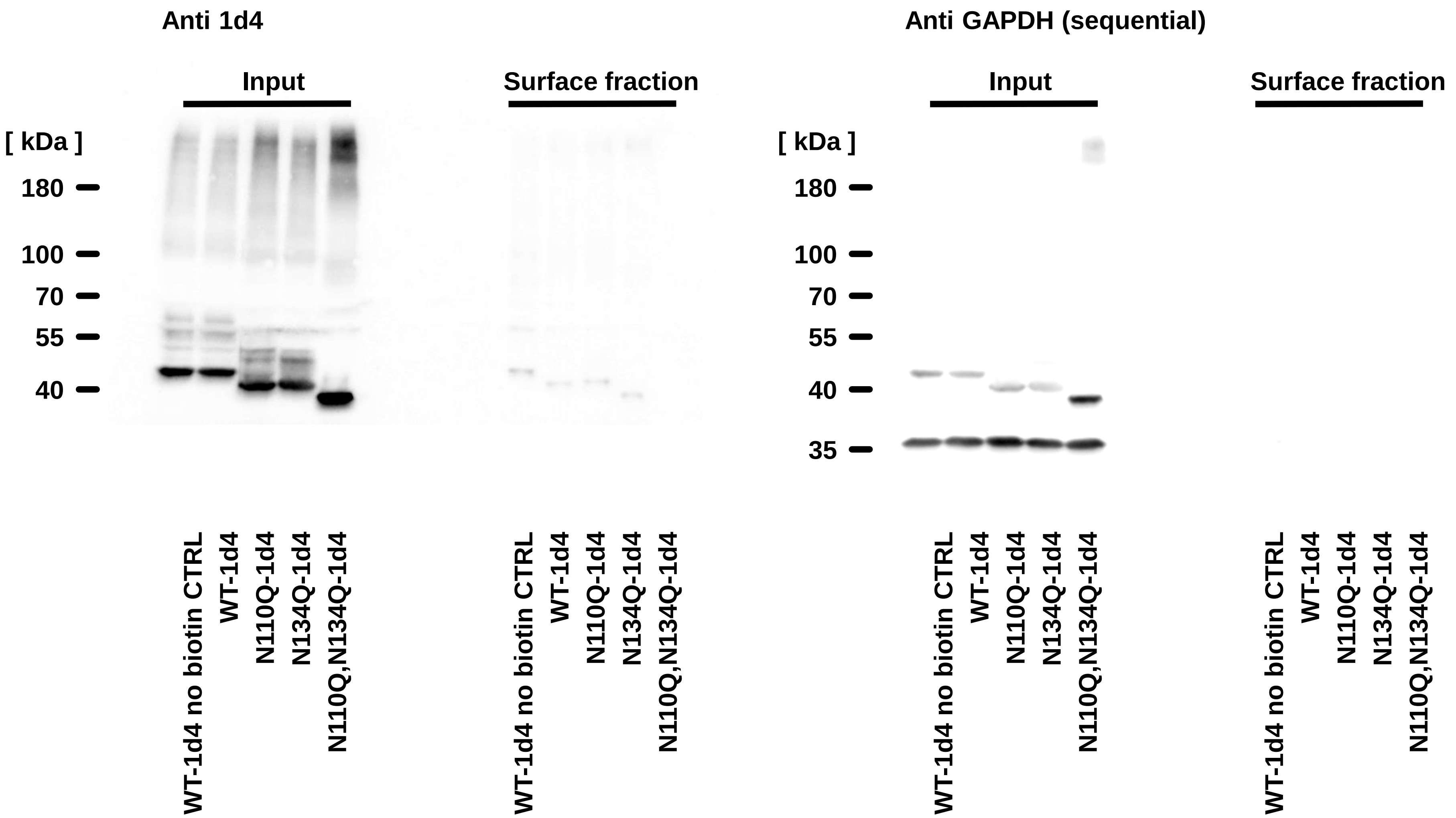

Supplementary figure 8

Western blot membrane 2: Surface expression (from figure 5b):

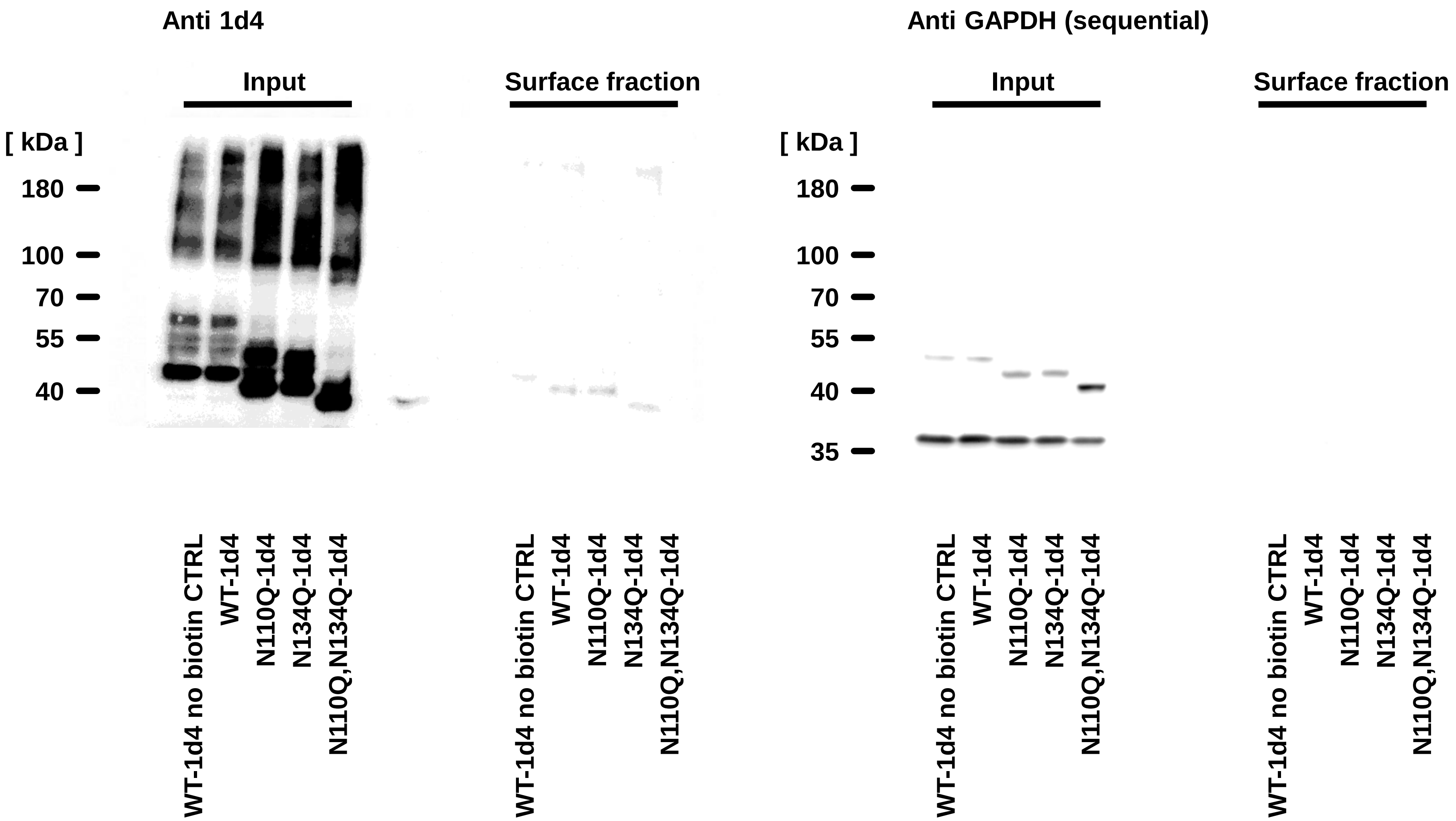

Supplementary figure 9

Western blot membrane 3: Surface expression (from figure 5b):

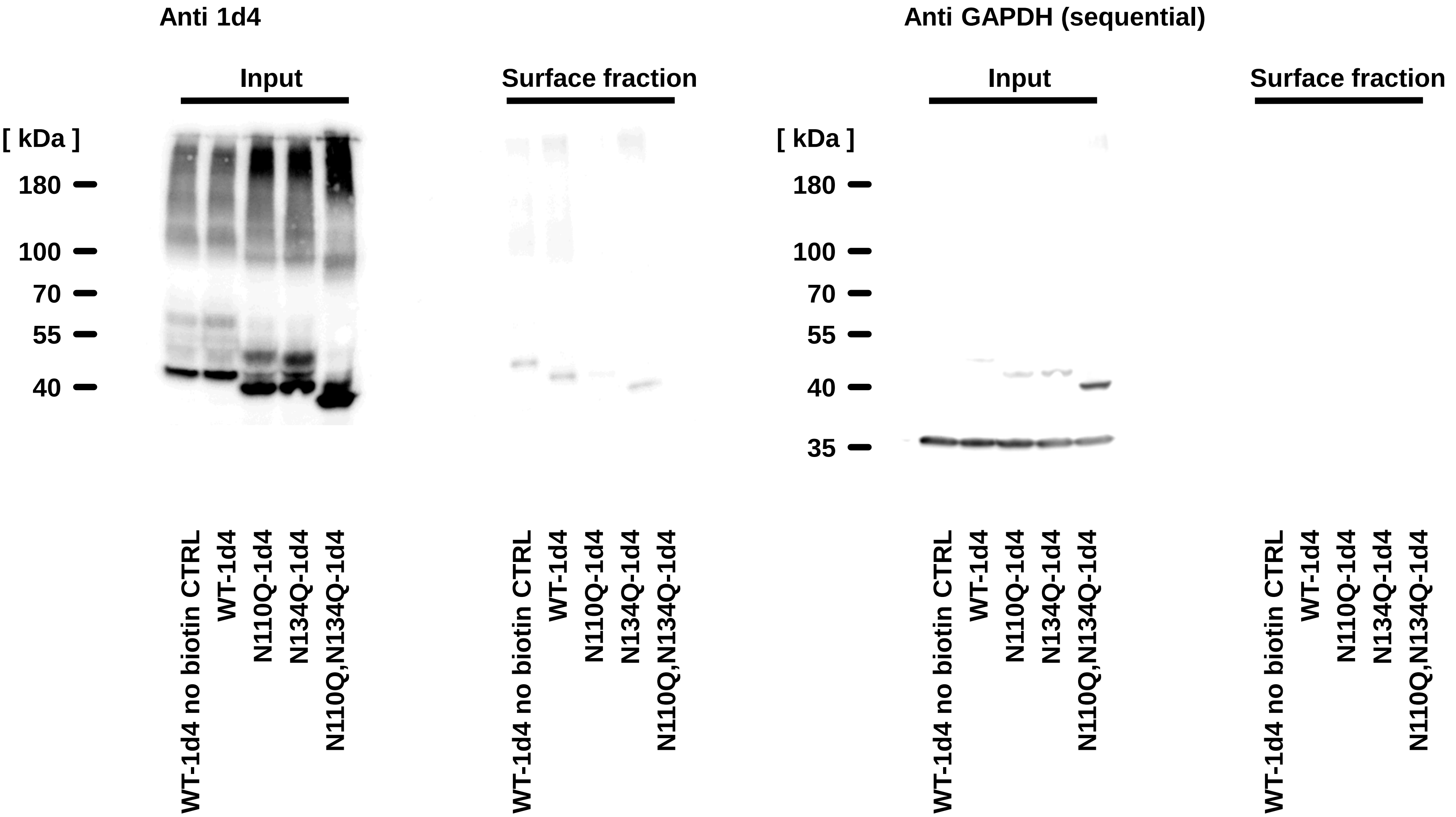

Supplementary figure 10

Western blot membrane: *O*-glycosylation of TREK-1 (from figure 6)

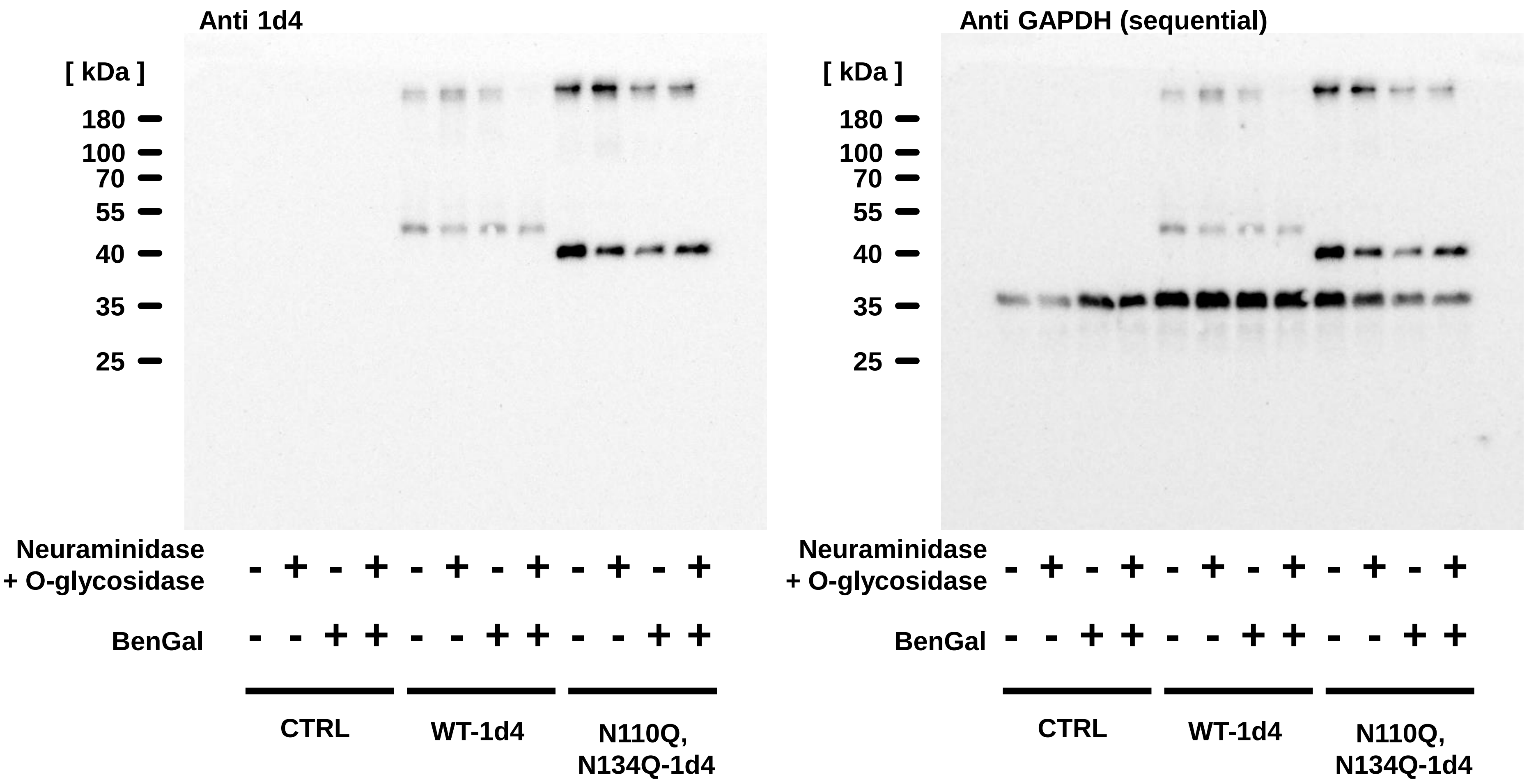

Supplementary figure 11

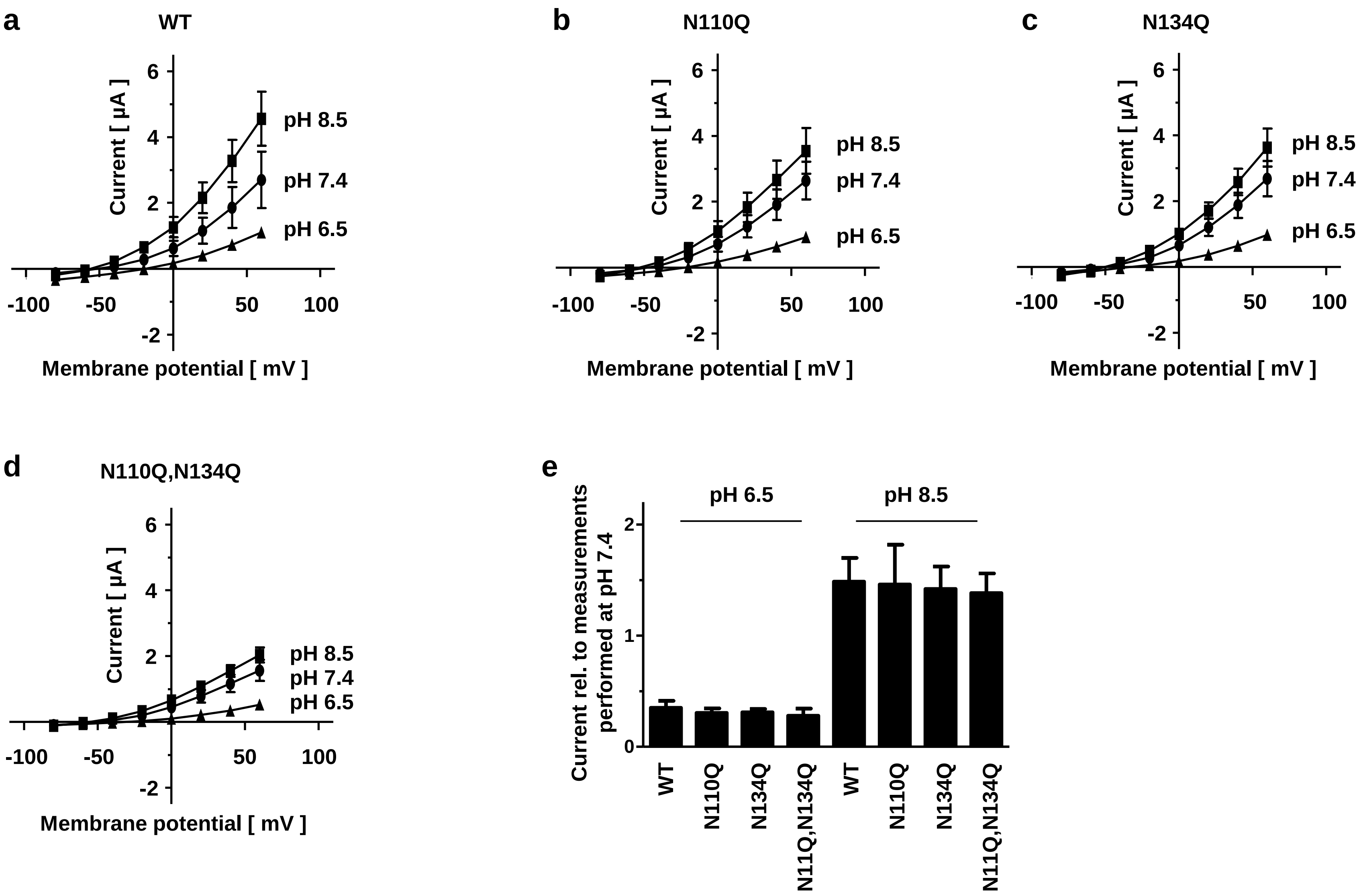

Supplementary figure 12

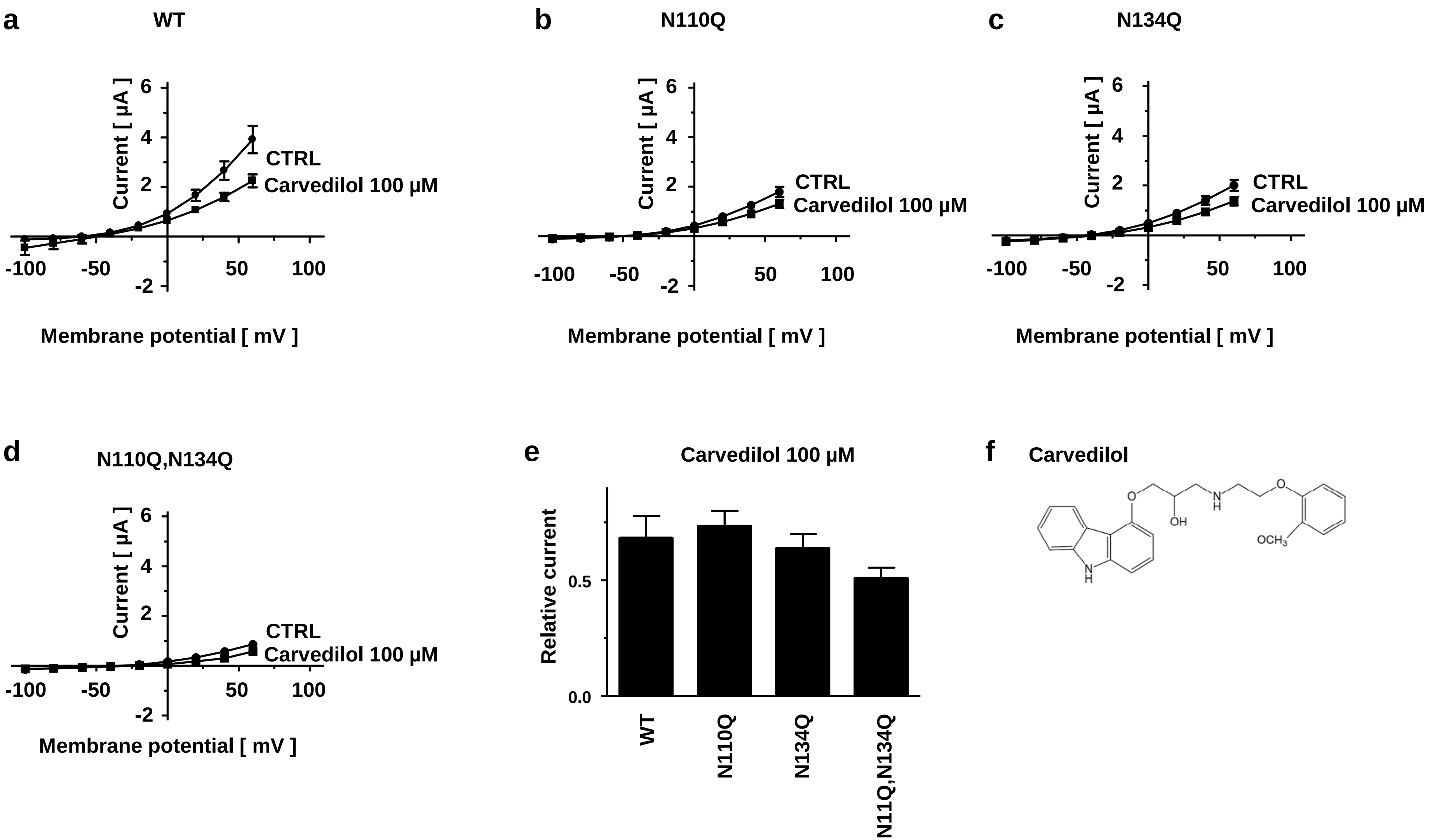

Supplementary figure 13

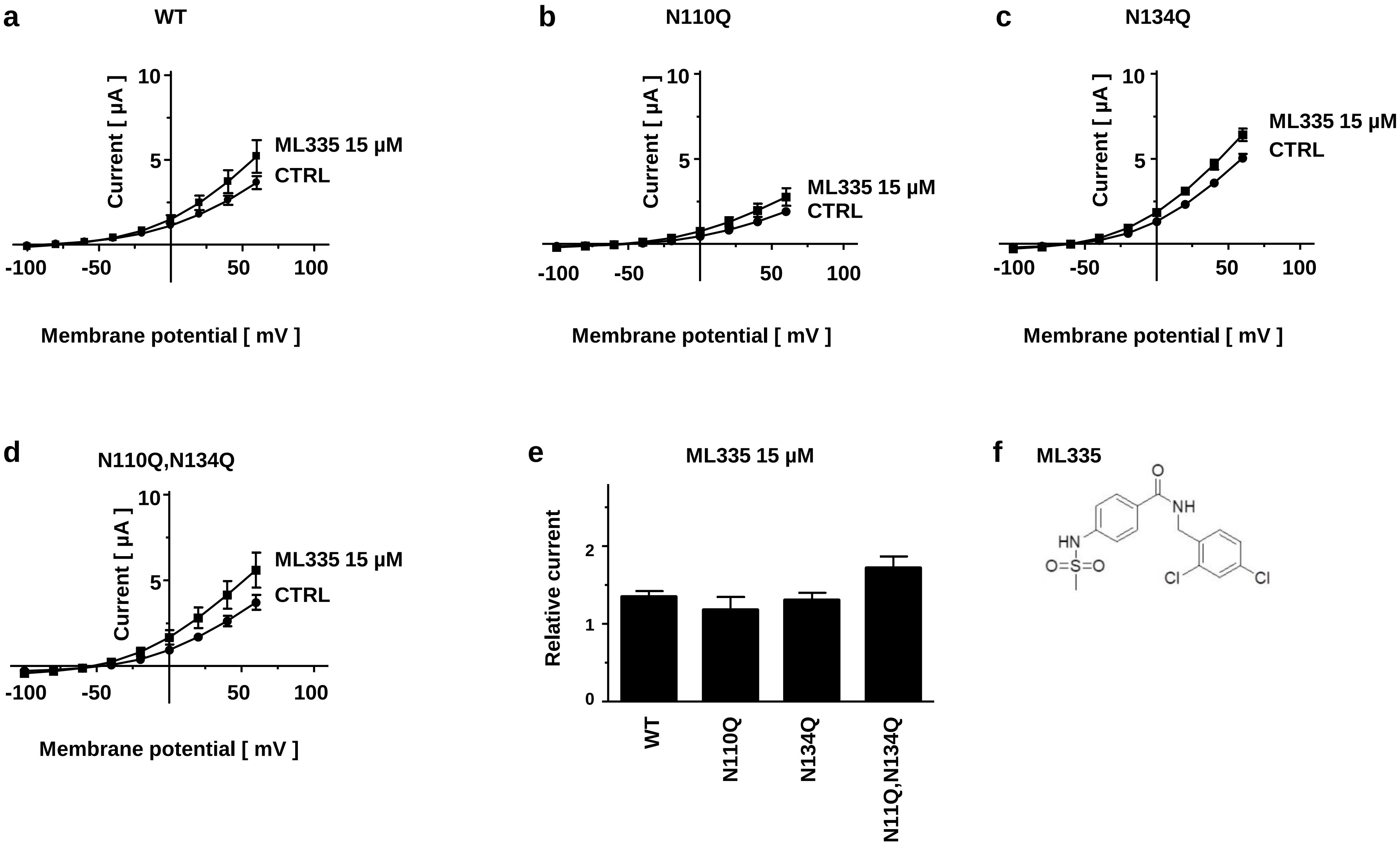

Supplement: Supplementary file 1 [file ijms-20-05193-s001.pdf]
